# Predictive Nomogram and Propensity Score Matching in Neuroendocrine Carcinoma of the Tubular Gastrointestinal Tract: A US Population-Based Clinical Outcome Study

**DOI:** 10.3390/cancers16111998

**Published:** 2024-05-24

**Authors:** Abdul Qahar Khan Yasinzai, Marjan Khan, Abdullah Chandasir, Diego Olavarria-Bernal, Amir Humza Sohail, Agha Wali, Bisma Tareen, Tena Nguyen, Ashley D. Fox, Aman Goyal, Israr Khan, Abdul Waheed, Asif Iqbal, Nabin Raj Karki, Kanak Das, Asad Ullah

**Affiliations:** 1 Department of Medicine, Bolan Medical College, Quetta 83700, Pakistan; abdul.qahar.aqk@gmail.com (A.Q.K.Y.); akwali092@gmail.com (A.W.); bismatareen@hotmail.com (B.T.); 2 Department of Medicine, Marshfield Clinic, Wisconsin, WI 54449, USA; khan.marjan@marshfieldclinic.org; 3 Medical College of Georgia, Augusta University, Augusta, GA 30912, USA; achandasir@augusta.edu (A.C.); tenguyen@augusta.edu (T.N.); 4 Department of Medicine, Texas Tech University Health Sciences Center, Lubbock, TX 79430, USA; diego.bernal@ttuhsc.edu; 5 Department of Surgery, University of New Mexico, Albuquerque, NM 87131, USA; ameer.hamzasohail@gmail.com; 6 Department of Hematology-Oncology, Augusta University, Augusta, GA 30912, USA; ashfox@augusta.edu; 7 Department of Medicine, Seth GS Medical College and KEM Hospital, Mumbai 400012, India; amanmgy@gmail.com; 8 Department of Medicine, Insight Hospital and Medical Center, Chicago, IL 60616, USA; israr.khan@insightchicago.com; 9 Department of Surgery, San Joaquin General Hospital, San Joaquin, CA 95231, USA; awaheed@sjgh.org; 10 Department of Medicine, Mercy Hospital, Ardmore, OK 73401, USA; asif702@gmail.com; 11 Division of Hematology-Oncology, University of South Alabama, Mobile, AL 36688, USA; nkarki@health.southalabama.edu; 12 Department of Medicine, Division of Gastroenterology, Texas Tech University Health Sciences Center, Lubbock, TX 79430, USA; kanak.das@ttuhsc.edu; 13 Department of Pathology, Texas Tech University Health Sciences Center, Lubbock, TX 79430, USA

**Keywords:** neuroendocrine carcinoma, SEER, chemotherapy, prognosis

## Abstract

**Simple Summary:**

Neuroendocrine carcinomas (NECs) of the tubular gastrointestinal tract are rare and associated with worse clinical outcomes. Data from 10,387 patients were collected from the SEER database with a median age of 63 years. The most common primary tumor site was the small intestine. women had a superior 5-year survival rate. Age > 65 years, descending colon and anorectal sites were associated with worse prognosis. Surgical intervention and tumors located in the small intestine and appendix showed a better prognosis.

**Abstract:**

**Background:** Neuroendocrine carcinomas (NECs) of the tubular gastrointestinal tract (GI-NECs) are rare and associated with worse clinical outcomes. This population-based study aims to highlight key demographics, clinicopathological factors, and survival outcomes in the US population. **Methods:** Data from 10,387 patients with GI-NECs were extracted from the Surveillance, Epidemiology, and End Result (SEER) database from 2000 to 2020. **Results:** Most patients were >40 years old at the time of presentation with a median age of 63 years old, with almost equal ethnic distribution per US population data. The most common primary tumor site was the small intestine (33.6%). The metastatic spread was localized in 34.8%, regional in 27.8%, and distant in 37.3% of cases, and the liver was the most common site of metastasis (19.9%) in known cases of metastases. Most NEC patients underwent surgery, presenting the highest 5-year overall survival of 73.2% with a 95% confidence interval (CI) (95% CI 72.0–74.4%), while chemotherapy alone had the lowest 5-year survival of 8.0% (95% CI 6.4–10.0%). Compared to men, women had a superior 5-year survival rate of 59.0% (95% CI 57.6–60.5%). On multivariate analysis, age > 65 (HR 2.49, 95% CI 2.36–2.54%, *p* ≤ 0.001), distant metastasis (HR 2.57, 95% CI 2.52–2.62%, *p* ≤ 0.001), tumor size > 4 mm (HR 1.98, 95%, CI 1.70–2.31%, *p* ≤ 0.001), esophageal (HR 1.49, 95% CI 0.86–2.58%, *p* ≤ 0.001), transverse colon (HR 1.95, 95% CI 1.15–3.33%, *p* ≤ 0.01), descending colon (HR 2.12, 95% CI 1.12, 3.97%, *p* = 0.02) anorectal sites, and liver or lung metastases were associated with worse survival. Surgical intervention and tumors located in the small intestine or appendix showed a better prognosis. **Conclusion:** GI-NECs are a group of rare malignancies associated with a poor prognosis. Therefore, epidemiological studies analyzing national databases may be the best alternative to have a more comprehensive understanding of this condition, assess the impact of current practices, and generate prognosis tools.

## 1. Introduction

Gastrointestinal neuroendocrine tumors (GI-NETs) are rare malignancies originating from multipotent stem cells located in the glands of the GI tract, with the capability to secrete biogenic amines and peptide hormones [1,2,3]. The reported incidence of GI-NETs in the United States is approximately 3.56 per 100,000 persons annually, with NECs composing approximately only 10–20% of all these tumors [4]. GI-NETs and NECs are characterized by the expression of neuroendocrine markers such as synaptophysin, chromogranin A, and somatostatin receptors [3,5]. However, GI-NETs can be differentiated based on their histological grade, differentiation, and location [1,2]. In 2019, the World Health Organization established classification guidelines for the malignant tumors of the digestive system, specifically noting the distinction between neuroendocrine tumors (NETs) as well-differentiated neoplasms and neuroendocrine carcinomas (NECs) as poorly differentiated neoplasms [2,6]. G3 staged NETs are considered well-differentiated yet high-grade tumors that are different/non-related to NECs [6]. However, the highly proliferative nature of G3 NETs has led to an increasing need for specific biomarkers to discern between G3 NETs and NECs [7,8].

GI-NECs can clinically present with nonspecific symptoms such as upper abdominal pain/discomfort or bleeding, whereas functional tumors may progress to present with secretory diarrhea, flushing, or multiple peptic ulcers [9]. Diagnostic imaging for NECs includes CTs or MRIs; however, advancements in endoscopic techniques have resulted in increased detection and diagnosis of GI-NECs [2,10]. The rarity of NECs has left much to be developed regarding the treatment and management of GI-NECs [11]. Currently, NECs are treated with multimodality therapy with small-cell lung cancer treatment protocols. With current NEC treatment regimens, prognosis is poor with survival ranging from 5 months in metastatic NECs to 38 months in the localized form of the disease [12]. In this study, we used the Surveillance, Epidemiology, and End Result (SEER) database to analyze demographic, treatment, and clinical outcomes, to further elucidate future therapeutic developments for GI-NECs. This study aims to provide a comprehensive understanding of GI-NECs, describe clinical patterns, assess the impact of current practices, and generate prognosis tools.

## 2. Materials and Methods

The data were extracted from the SEER database between the years 2000 and 2020 using SEER Stat software (Version 8.4.2). Inclusion criteria included histologically confirmed gastrointestinal neuroendocrine tumor/neoplasm, international classification of diseases version 3 (ICD-O-3) (https://seer.cancer.gov/seerstat/releasenotes.html, accessed 15 November 2023). Patients without histological confirmation and tumors classified as in situ were excluded. Extracted variables included age, gender, race, tumor stage, tumor grade, tumor histology, tumor size, lymph node status, type of treatment received, and survival. Statistical analysis was performed using R version 4.2.3 data-analysis software. Endpoints examined included overall survival, mortality, and 1- and 5-year cancer-specific survival. Univariate analysis was conducted to identify significant factors for multivariate analysis. The Cox regression method was used to calculate hazard ratios and to identify independent factors on survival outcomes. Unidentified or missing data were removed from multivariate analysis to ensure validity and remove biases in the case of non-random missing data. The data was analyzed using multivariate Cox regression with statistical significance defined as *p* < 0.05. A prognostic nomogram was also constructed to be used to predict future patient mortality and survival rate. This was done through the “rms” package in R. After pertinent prognostic variables were identified, the resultant mathematical models were then used to construct a nomogram. All variables that were found to have significance on the survival of patients through Cox Regression were added to the prognostic nomogram. The nomogram was then built using a Cox proportional hazards method. By giving each variable, a numerical score determined by how much of an impact it has on the overall forecast, the nomogram provides a visual representation of the predictive model. Following the addition of these scores, a total point value is produced that is correlated with the expected likelihood or risk of the specified outcome, such as the course of the disease or survival. The correctness and generalizability of the nomogram were confirmed through the use of internal and external validation approaches. Internal validation entailed evaluating the nomogram’s performance within the original dataset using resampling techniques like bootstrapping or cross-validation.

## 3. Results

Data from 10,387 patients with GI-NECs of the tubular GI tract met the inclusion criteria.

### 3.1. Demographic Characteristics

The distribution by gender in patients with GI NEC was relatively similar with a slight overall male predominance (51.5%). The median age of the patients was 63 years old, with ages ranging from 6 years old to older than 90 years of age. The highest number of patients were between 60 and 69 years of age (25.7%). White patients accounted for a majority (65.8%) of cases, with Black patients having the second most cases (14.4%). The data show an almost equal racial/ethnic distribution through the US population (https://www.census.gov/quickfacts/fact/table/US/PST045223, accessed on 14 April 2024). The breakdown of the median annual household income was close to even, with a slight majority (51.8%) of patients earning below $70,000 per year. A majority of our patients were from metropolitan areas (87.0%) (Table 1) (Supplemental Figure S1).

### 3.2. Tumor Characteristics and Treatment Modalities:

**Figure 1:**
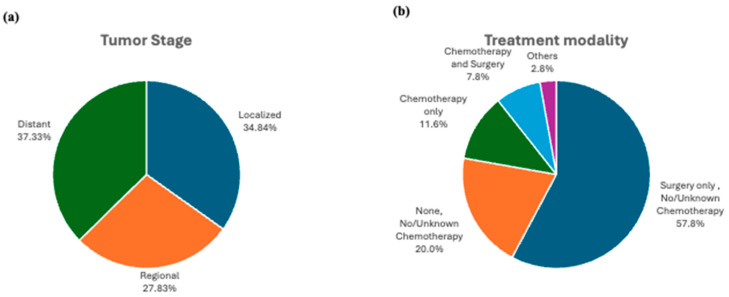
Distribution of tumor stage (**a**) and treatment modality (**b**) in GI-NEC cases.

Most patients were younger than 65 years old, with a slight predominance in men (51.5%). The most common site for the NECs was the small intestine (33.6%), followed by the rectum (18.1%) and the stomach (15.4%). The largest proportion of patients had tumors smaller than 2 cm (44.5%). The most common tumor stage was distant (37.3%) followed by localized (34.8%) (Figure 1a).

**Figure 2:**
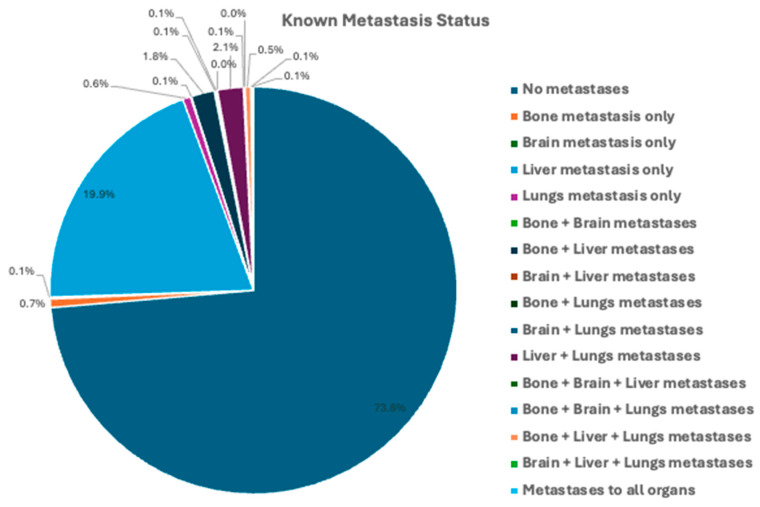
Regional lymph node status and distant metastasis at time of diagnosis for 10,387 patients with neuroendocrine carcinomas of the tubular GI tract from the Surveillance, Epidemiology, and End Results (SEER) database (2000–2020).

The most common metastasis was to the liver (19.9%). The majority of patients (73.6%) with known metastasis information had no metastases. Lymph node status was known in 4391 (42.3%) of patients. In patients with known lymph node status, 3338 (76.0%) of patients had positive regional lymph nodes (Figure 2).

The most common treatment modality for patients with GI-NEC was surgery only (57.8%), followed by chemotherapy only (11.6%). A significant minority of patients did not receive any treatment (20.0%). A total of 210 patients with GI-NEC (2.0%) received multimodal therapy, or a treatment regimen consisting of chemotherapy, radiation therapy, and surgery (Figure 1b).

### 3.3. Overall and Cause-Specific Survival of Patients with GI-NEC

**Figure 3:**
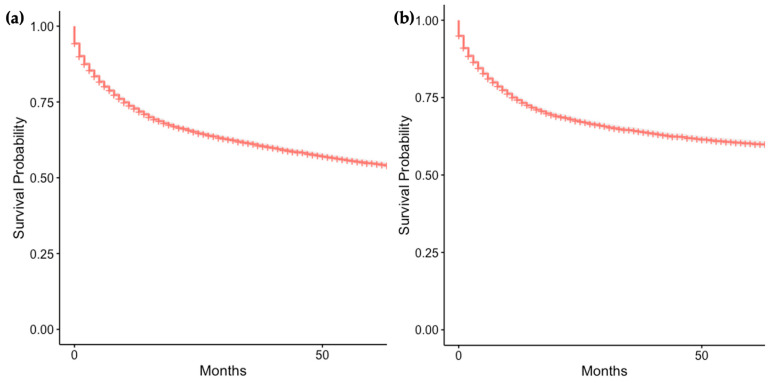
Kaplan–Meier curve of overall survival (**a**) and cause-specific survival (**b**) of GI-NEC cases.

The 1-year and 5-year overall survival of the patient group was 72.9% (95% CI 72.0–73.7%) and 54.7% (95% CI 53.7–55.7%), respectively (Figure 3a). SEER calls disease-specific survival “cause-specific survival” in their database. The 1-year and 5-year cause-specific survival of the study group was 74.2% (95% CI 73.3–75.2%) and 60.0% (95% CI 59.0–61.1%) (Figure 3b) (Supplemental Figure S1).

### 3.4. Survival Analysis of Demographic Factors

#### 3.4.1. Outcomes by Age

**Figure 4:**
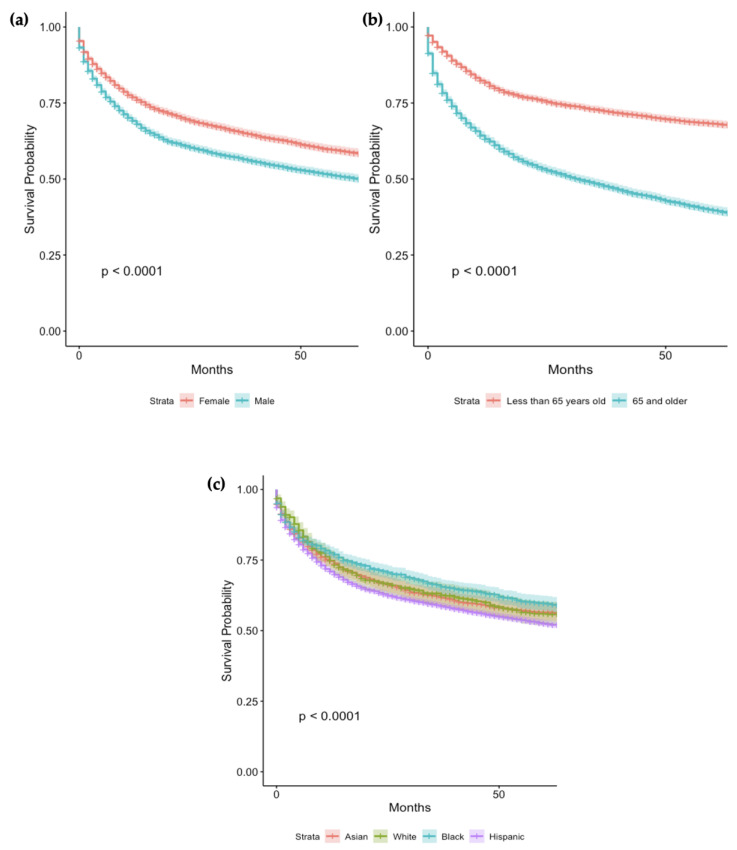
Survival Analysis of GI-NEC cases based on gender (**a**), age (**b**) and race (**c**).

The 1-year and 5-year survival of patients below the age of 65 years was 81.7% (95% CI (80.7–82.8%) and 68.2% (95% CI 66.9–69.5%), respectively. The 1-year and 5-year survival of patients 65 years of age and older was 62.4% (95% CI 61.0–63.8%) and 38.9% (95% CI 37.4–40.4%), respectively (Figure 1a) (Supplemental Table S4). Univariate analysis determined older age to be associated with an increased mortality (H.R = 2.62, 95% CI 2.47–2.77%, *p* < 0.001) (Figure 4b).

#### 3.4.2. Outcomes by Sex

The 1-year and 5-year survival of female patients was 76.9% (95% CI 75.7–78.1%) and 59.0% (95% CI 57.6–60.5%), respectively (Supplemental Table S2). The 1-year and 5-year survival of male patients was 69.1% (95% CI 67.9–70.4%) and 50.7% (95% CI 49.3–52.1%), respectively (Figure 1b). Univariate analysis found males to have a higher mortality rate (H.R = 1.28, 95% CI 1.21–1.35%, *p* < 0.001) (Figure 4a).

#### 3.4.3. Outcomes by Race

The 1-year and 5-year survival for white patients was 70.9% (95% CI 69.8–72.0%) and 52.6% (95% CI 51.3–53.8%), respectively. The 1-year and 5-year survival for black patients was 77.6% (95% CI 75.5–79.8%) and 59.7% (95% CI 57.1–62.4%), respectively. The 1-year and 5-year survival for Hispanic patients was 74.7% (95% CI 72.3–77.2%) and 56.5% (95% CI 52.6–59.5%). The 1-year and 5-year survival for Asian patients was 74.8% (95% CI 71.3–78.4%) and 56.0% (95% CI 51.9–60.4%) (Supplemental Table S3) (Figure 4c). Univariate analysis found white patients to have a lower survival rate (H.R = 1.17, 95% CI 1.07–1.28%, *p* < 0.001) (Figure 4c).

### 3.5. Survival Analysis of Tumor Characteristics and Treatment ModalitiesTumor Characteristics

#### Tumor Characteristics

**Figure 5:**
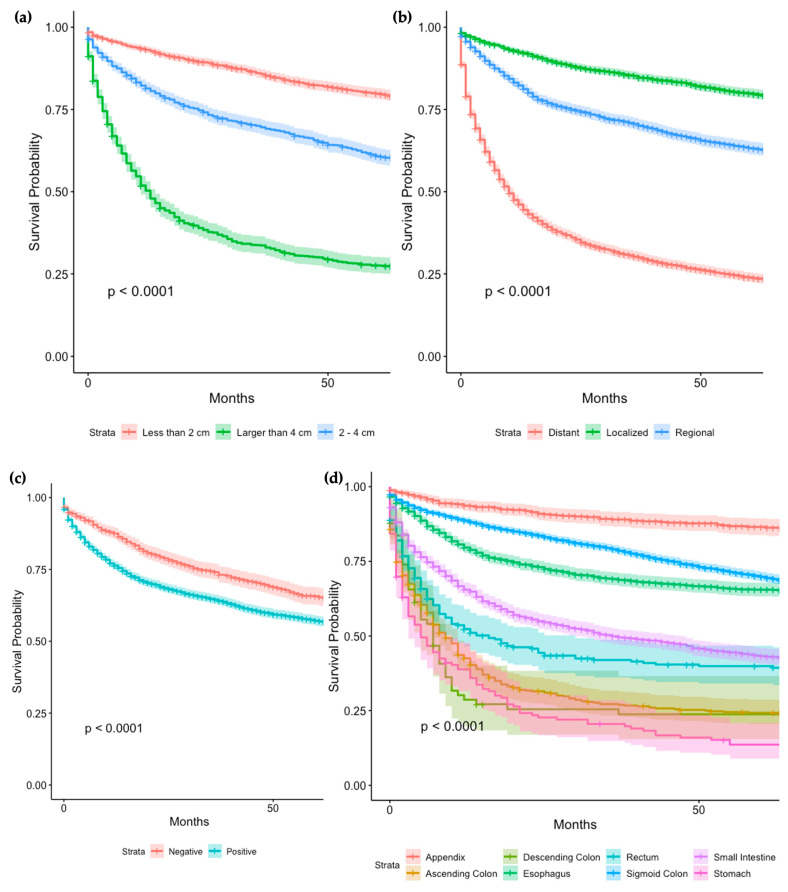
Survival Analysis of GINEC cases based on tumor size (**a**), tumor stage (**b**) and regional lymph node status (**c**), survival analysis of GINEC cases based on tumor site (**d**).

The survival rates for GI-NEC vary across different gastrointestinal sites. Notably, GI-NEC of the appendix demonstrated the best survival rates with 1-year and 5-year survivals of 93.7% (95% CI 91.8–95.5%) and 86.5% (95% CI 83.8–89.4%), respectively (Supplemental Table S7). Univariate analysis revealed significantly worse prognoses for GI-NEC of the ascending colon (H.R = 3.56, 95% CI 3.17–4.00%, *p* < 0.001), descending colon (H.R = 4.16, 95% CI 3.20–5.42%, *p* < 0.001), esophagus (H.R = 5.98, 95% CI 5.24–6.81%, *p* < 0.001), sigmoid colon (H.R = 2.44, 95% CI 2.07–2.86%, *p* < 0.001), stomach (H.R = 2.02, 95% CI 1.86–2.20%, *p* < 0.001), and transverse colon (H.R = 4.99, 95% CI 4.17–5.97%, *p* < 0.001) compared to GI-NEC of the small intestine, while GI-NEC of the appendix exhibited a better survival rate (H.R = 0.34, 95% CI 0.28–0.42%, *p* < 0.001) (Figure 5d).

The 5-year survival rates for patients with localized, regional, and distant tumors were 79.9% (95% CI 78.4–81.4%), 63.2% (95% CI 61.3–65.2%) and 24.0% (95% CI 22.5–25.5%), respectively (Supplemental Table S5). Localized tumors were associated with a better prognosis (HR = 0.51, *p* < 0.001), while distant tumors had a worse prognosis (HR = 3.05, *p* < 0.001) compared to regional tumors (Figure 5b).

For tumors smaller than 2 cm, the survival rate at 5 years was 79.8% (95% CI 78.2–81.5%). Tumors measuring 2 cm to 4 cm had survival rates of 60.8% (95% CI 58.5–63.3%), while those greater than 4 cm had rates of 27.6% (95% CI 25.2–30.2%) (Supplemental Table S6). Larger tumors were linked to higher mortality, with tumors > 4 cm showing the poorest outcomes (HR = 5.20, *p* < 0.001) (Figure 5a).

The 5-year survival rates of patients with negative and positive regional lymph node statuses were 65.7% (95% CI 62.7–68.8%) and 57.2% (95% CI 55.4–58.9%), respectively (Supplemental Table S8). Positive lymph node status was associated with increased mortality (HR = 1.33, *p* < 0.001) (Figure 5c).

### 3.6. Treatment Modalities

**Figure 6:**
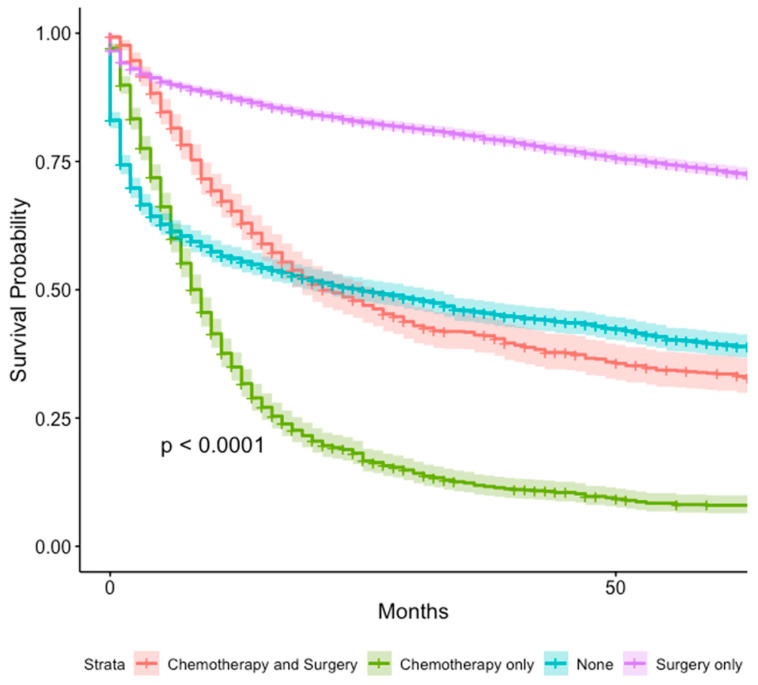
Survival analysis of GI-NEC cases based on treatment modality.

The 1-year and 5-year survival for patients that received chemotherapy only was 34.9% (95% CI 32.3–37.8%) and 8.0% (95% CI 6.4–10.0%). The 1-year and 5-year survival for patients that received surgery only was 87.3% (95% CI 86.5–88.2%) and 73.2% (95% CI 72.0–74.4%). The 1-year and 5-year survival for patients who received chemotherapy and surgery was 65.3% (95% CI 62.1–68.7%) and 33.6% (95% CI 30.3–37.2%). The 1-year and 5-year survival for patients without treatment was 56.1% (95% CI 54.0–58.3%) and 33.6% (95% CI 30.3–37.2%) (Supplemental Table S9). Univariate analysis found patients who received chemotherapy only had the worst outcomes (H.R = 6.02, 95% CI 5.57–6.51%, *p* < 0.001) (Figure 6). Other treatment modalities were not analyzed due to low power.

### 3.7. Multivariate Analysis

**Figure 7:**
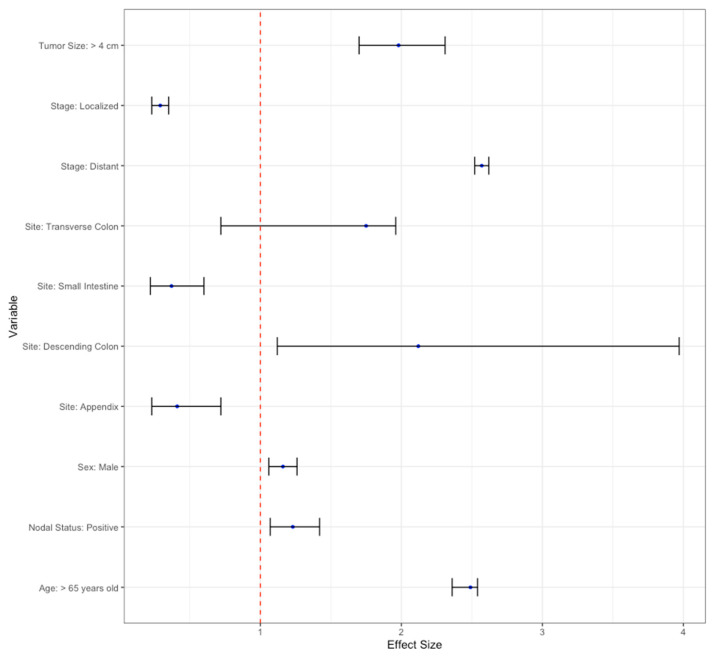
Forest plot depicting hazard ratios of variables that impact mortality of patients with GI-NEC.

Multivariable analysis was performed using Cox Survival Regression analysis. Independent factors associated with increased mortality were identified as age greater than 65 years, male gender, a GI-NEC tumor site of the transverse colon or descending colon, tumor stage of distant, positive nodal status, or a tumor size of >4 cm. Factors associated with decreased mortality were a tumor site of an appendix or small intestine, or a localized tumor (Table 2). A hazard ratio above 1 indicates a higher incidence of mortality in the pertaining group, whereas a hazard ratio below 1 indicates a decreased incidence of mortality. All significant statistical factors and their hazard ratios are displayed in the Forest plot. The hazard ratios are displayed along with confidence intervals (Figure 7).

### 3.8. Prognostic Nomogram

**Figure 8:**
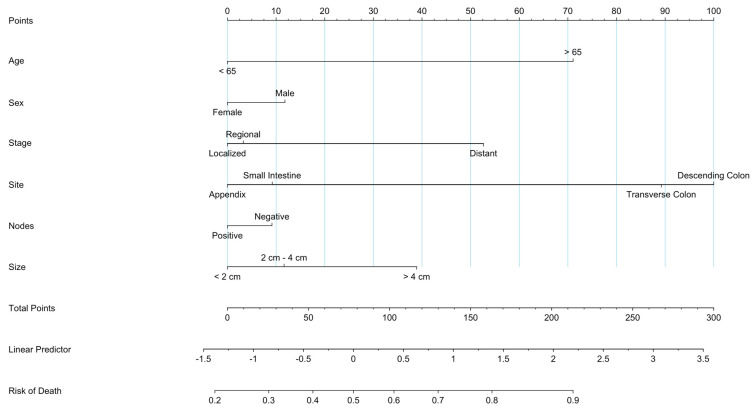
Prognostic nomogram predicting mortality for patients with GINEC.

To create a predictive measure that could extrapolate the survival of patients with GI-NEC, a prognostic nomogram was created to measure patient survivability using the factors of age, treatment, and systemic treatment. The nomogram visually represents the predictive model by assigning a numerical score to each variable based on its relative contribution to the overall prognosis. These scores are then summed to generate a total point value, which correlates with the predicted probability or risk of the defined outcome, which was overall survival (OS). The prognostic nomogram showed increased mortality for patients with a higher age. In addition to that, the prognostic nomogram also predicted increased mortality for patients who had tumors greater than 4 cm in size. Distantly metastasized tumors were associated with a significantly increased probability of death, whereas regional metastases were associated with a moderate increase in mortality. The nomogram also showed a slight increase in mortality for patients that were males. Tumors of the descending or transverse colon were associated with higher mortality, whereas tumors of the small intestine and appendix were associated with decreased mortality (Figure 8).

### 3.9. Propensity Score Matching

To further investigate the effect of gender on mortality, propensity score matching was performed. Male and Female patients were matched based on the demographic, tumor, and treatment factors that were found to have significant effect on survival based on the multivariate analysis—age, tumor stage, tumor size, tumor site, and regional lymph node status. Propensity score matching showed that when matched to females of similar age, tumor stage, size, site and regional lymph node status, males had a higher mortality rate (*p* = 0.049).

## 4. Discussion

Our study analyzed data from 10,387 patients, most of whom were white, finding a slight predominance of GI NEC in the male population. The small intestine was the most common site, and the most common stage was distant (37.3%). The liver is the most frequent site of metastasis. Surgery was the most common treatment modality.

The univariate analysis showed that older age is associated with increased mortality. Factors associated with worse prognosis: tumors of the esophagus, stomach, and at any part of the colon. Tumor size > 4 cm. On the other hand, tumor of the appendix and patient who underwent only surgery showed the best survival rate.

The multivariable analysis identified factors associated with increased mortality: age greater than 65 years, male gender, a GI-NEC tumor site of the transverse colon or descending colon, tumor stage of distant, positive nodal status, or a tumor size of >4 cm. Factors associated with decreased mortality were a tumor site of the appendix or small intestine or a localized tumor.

With all this information, we created a prognostic nomogram, which showed increased mortality for older age, patients with tumors greater than 4 cm in size, distant metastasis, and tumors of the descending or transverse colon. On the other hand, tumors of the small intestine and appendix were associated with decreased mortality. Men present a slight increase in mortality, which was confirmed by the propensity score analysis.

Of all GI neuroendocrine malignancies, GI-NECs represent 10–20% of these cancers [13]. However, due to their aggressive nature, there are limited studies investigating the epidemiology of GI-NECs as most cases are commonly diagnosed in their advanced stages [13,14].

A previous SEER database study identified 5509 cases of GI-NECs between 2000 and 2012, with most NECs occurring in the colon (26%), pancreas (20%), and rectum (12%) [15]. In our study, the most common site was the small intestine. This difference may be attributed to the inclusion of pancreatic NEC in the previous study, while our excluded and included SEER data through the year 2018. Moreover, GI-NEC site variations may depend on geography; in Taiwan and Japan, the rectum is the most common primary site, while in Australia and Canada, the small intestine is the most common site, similar to our study [16,17,18,19,20]. The common site of metastasis is strongly predicted by the primary site of tumor [21]. Because we cannot discern if these differences can be attributed to genetic or environmental factors, further studies are needed to substantiate the existence of possible modifiable GI-NEC risk factors.

Furthermore, in our study, older age (>65 years) at diagnosis, size >4 cm, and distant metastasis were independent negative risk factors affecting overall survival. Tumors of the esophagus, anus, cecum, and colon were also risk factors associated with increased mortality. NEC of the colon has been found to have worse survival compared to other sites of the GI tract [22]. Colon NEC have a higher tendency to metastasize than their peers contributing to its worse survival [23].

Age as an independent risk factor was also found in a previous study where the authors analyzed data of 1861 patients, between 2004 and 2013. They found that younger age (<65 years) correlates with improved survival (*p* < 0.001) [24].

Sorbye et al. demonstrated that patients with primary colon tumors had significantly shorter median survival compared with other primary tumors [25]. Regarding independent factors associate with decreased mortality, a previous study also reported GI NEC from the small intestine as presenting better 5-year OS (68.2%) compared NECs of the rectum or colon (*p* < 0.001) [26].

Regarding treatment outcomes, our study found no added survival benefit with the addition of chemotherapy and radiation compared to surgical resection alone, but information about adjuvant therapy was limited.

In this regard, the Expert Consensus Practice Recommendations of the North American Neuroendocrine Tumor Society for the management of high-grade gastroenteropancreatic and gynecologic neuroendocrine published in 2023 states that the role of surgery, even with locoregional disease, is questionable due to poor prognosis, and suggest systemic therapy with chemoradiation especially in cases where surgery represents higher morbidity. The stronger indication of surgery is for localized NEC, but even here, there is an indication for chemotherapy and possibly radiation (esophageal, anal some rectal tumors). The same publication states chemoradiation strategies may provide durable local control for primary and metastatic disease [27]. There is still room for discoveries and approaches regarding the best treatment. Currently, there is an open trial to evaluate the efficacy of neoadjuvant chemotherapy in terms of DFS in patients with localized tumors [28].

The molecular landscape in NENs varies according to the type and location of the tumor [29]. Previous studies have highlighted the molecular complexities between well-differentiated G3 GI-NETs and poorly differentiated GI-NECs, paving the way for new treatment and management avenues [30]. Well-differentiated GI-NETs have a low rate of genomic alterations with a tumor mutational burden average of 1.09 mutations per megabase [31]. Frequent alterations are *ARID1A*, *ATM*, *CDKN2A*, *KDM5A*, *ESR1*, *MYC*, *RB1*, *MEN1*, and *ATRX* [32,33]. In contrast, NECs have intermediate tumor mutational burden with an average of 5.45/Mb and frequent alterations in *TP53*, *KRAS*, *RB1*, *CSMD3*, *APC*, *CSMD1*, *LRATD2*, *TRRAP*, and *MYC* [32,34].

*APC*, *TP53*, *KRAS*, or *BRAF* mutations occurred in 83% of large cell (LC) NECs and only 21% of G3-NECs, with a positive predictive value of about 83%. The study also revealed differences in microsatellite instability (MSI) between GI-NEC (5.3%) and G3 GI-NETs (3.4%) [32].

Based on Platinium-etoposide (PE) regimens outlined for SCLC, GI-NECs have a worse prognosis than their G3 GI-NET counterparts, with a median overall survival of <1 year with treatment and an overall response rate (ORR) of 30–56% [12,30,35]. On the other hand, G3 GI-NETs have a better overall prognosis but an inferior response to combination (ORR from 0% to 17%) [35].

The drastic differences in prognosis and treatment responses between G3 GI-NETs and GI-NECs solidify the need to elucidate the genomic differences between the two cancers.

### Limitations

A major limitation of our study is the changing classification of NECs over time; the current WHO classification cannot be directly extrapolated to the categories available from the SEER database. Regarding therapy, the timing of the chemotherapy (adjuvant vs. neoadjuvant), the regimens and the duration of therapy are not available from the SEER database. Clinical factors like socioeconomic status, comorbidities, and side effects from therapies that might affect the outcomes are not always accurately coded or cannot be obtained from the SEER database. In addition, the prognostic nomogram was not tested prospectively. Despite these limitations, our study attempts to further characterize clinical and demographical aspects of the rare neuroendocrine carcinomas of the tubular GI tract.

## 5. Conclusions

To our knowledge, this is the largest population database study to date on GI-NECs of the tubular GI tract. The most common sites of NECs were the small intestine. Affected adults were generally older than 40 years old with the highest peak between 60 and 69 years old, with a small male predominance. In addition, compared to men, women had a better overall 5-year survival rate. Multivariate analysis shows older age (>60 years old), tumor size > 4 cm, and distant metastasis as independent risk factors associated with mortality. Esophageal, colonic, and anorectal locations of GI-NECs are also indicative of increased mortality, along with the presence of liver or lung metastases. Surgical resection provided the best outcome with no clear benefit observed for radiation and chemotherapy. However, our analysis also indicated better prognosis-associated tumor sites in the small intestine and appendix. This study provides better clinical and demographic insight into future clinical trials to include patients from all ethnicities and races for future personalized therapeutic approaches.

## Figures and Tables

**Table 1:** Demographic profiles, tumor size, and staging of 10,387 patients with gastrointestinal neuroendocrine carcinoma from the Surveillance, Epidemiology, and End Results (SEER) database, 2000–2020.

Variable (n = 10,387)		Frequency (%)
Age	<65 years	5652 (54.4%)
	≥65 years	4735 (45.6%)
Gender	Female	5034 (48.4%)
	Male	5353 (51.5%)
Ethnicity	White	6832 (65.8%)
	Black	1500 (14.4%)
	Hispanic	1276 (12.3%)
	Asian or Pacific Islander	608 (5.9%)
	American Indian or Alaska Native	56 (0.5%)
	Unknown	115 (1.1%)
Site	Esophagus	315 (3.0%)
	Stomach	1599 (15.4%)
	Small Intestine	3489 (33.6%)
	Cecum	772 (7.4%)
	Anus	137 (1.3%)
	Appendix	731 (7.0%)
	Ascending colon	467 (4.5%)
	Hepatic flexure	109 (1.0%)
	Transverse colon	146 (1.4%)
	Splenic flexure	48 (0.5%)
	Descending colon	74 (0.7%)
	Sigmoid colon	251 (2.4%)
	Rectosigmoid junction	193 (1.9%)
	Rectum	1885 (18.1%)
	Large intestine, NOS	171 (1.6%)
Size	Known	5111 (49.2%)
	Unknown	5276 (80.8%)
	Size where known (n = 5327)	
	<2 cm	2274 (44.5%)
	2–4 cm	1590 (31.1%)
	>4 cm	1247 (24.4%)
Stage	Known	9153 (88.1%)
	Unknown	1234 (11.9%)
	Stage where known (n = 9153)	
	Localized	3189 (34.8%)
	Regional	2547 (27.8%)
	Distant	3417 (37.3%)
Lymph node status	Known	4391 (42.3%)
	Unknown	5996 (57.7%)
	Lymph node status where known (n = 4391)	
	Positive	3338 (76.0%)
	Negative	1053 (24.0%)
Median Annual Household Income	<$70,000	5378 (51.8%)
	≥$70,000	5007 (48.2%)
Housing Location	Metropolitan	9032 (87.0%)
	Rural	1347 (13.0%)

**Table 2:** Multivariate analysis of GINEC variables.

		Univariate		Multivariate	
Variables		Hazard Ratio (95% C.I)	*p*-Value	Hazard Ratio (95% C.I)	*p*-Value
Age	<65 years old	1 (reference)	1 (reference)	1 (reference)	1 (reference)
	≥65 years old	1.62 (2.47–2.77)	<0.001	2.49 (2.36–2.54)	<0.001 *
Sex	Male	1.28 (1.21–1.35)	<0.001	1.16 (1.06–1.26)	<0.001 *
	Female	1 (reference)	1 (reference)	1 (reference)	1 (reference)
Race	White	1.17 (1.07–1.28)	<0.001	0.99 (0.85–1.16)	0.91
Site	Esophagus	5.98 (5.24–6.81)	<0.001	1.49 (0.86–2.58)	0.15
	Small intestine	0.21 (0.11–0.31)	<0.001	0.37 (0.22–0.60)	<0.001 **
	Ascending Colon	3.56 (3.17–4.00)	<0.001	1.19 (0.72–1.96)	0.49
	Transverse Colon	4.99 (4.17–5.97)	<0.001	1.95 (1.15–3.33)	0.01 *
	Descending Colon	4.16 (3.20–5.42)	<0.001	2.12 (1.12–3.97)	0.02 *
	Sigmoid Colon	2.44 (2.07–2.86)	<0.001	1.41 (0.83–2.41)	0.2
	Appendix	0.29 (0.18–0.40)	<0.001	0.41(0.23–0.72)	0.002 **
	Stomach	2.02 (1.86–2.20)	<0.001	1.12 (0.68–1.84)	0.39
Stage	Localized	0.51 (0.46–0.55)	<0.001	0.29 (0.23–0.35)	<0.001 **
	Regional	1 (reference)	1 (reference)	1 (reference)	1 (reference)
	Distant	3.05 (2.85–3.27)	<0.001	2.57 (2.52–2.62)	<0.001 *
Nodal Status	Negative	1 (reference)	1 (reference)	1 (reference)	1 (reference)
	Positive	1.33 (1.20–1.48)	<0.001	1.23 (1.07–1.42)	0.003 *
Tumor Size	<2 cm	1 (reference)	1 (reference)	1 (reference)	1 (reference)
	2 cm–4 cm	2.08 (1.87–2.30)	<0.001	1.14 (0.99–1.31)	0.07
	>4 cm	5.20 (4.71–5.75)	<0.001	1.98 (1.70–2.31)	<0.001 *
Treatment	Chemotherapy and Surgery	1 (reference)	1 (reference)	1 (reference)	1 (reference)
	Chemotherapy only	6.02 (5.57–6.51)	<0.001	1.45 (0.75–4.19)	0.68
	None	1.07 (0.97–1.18)	0.2	0.94 (0.11–7.80)	0.96
	Surgery only	0.36 (0.33–0.39)	<0.001	0.35 (0.05–2.74)	0.32

* = associated with increased mortality; ** = associated with decreased mortality.

## Data Availability

All data are publicly available. The data of this manuscript were presented at the Society of American Gastrointestinal and Endoscopic Surgeons (SAGES), Las Vegas, Nevada, 31 August–3 September 2021.

## Supplementary Materials

The following supporting information can be downloaded at: https://www.mdpi.com/article/10.3390/cancers16111998/s1, Table S1: Overall Survival and Cause Specific Survival Percentages; Table S2: 1-year and 5-year Survival Percentages by Gender; Table S3: 1-year and 5-year Survival Percentages by Race; Table S4: 1-year and 5-year Survival Percentages by Age; Table S5: 1-year and 5-year Survival Percentages by Tumor Stage; Table S6: 1-year and 5-year Survival Percentages by Tumor Size; Table S7: 1-year and 5-year Survival Percentages by Tumor Site; Table S8: 1-year and 5-year Survival Percentages by Regional Lymph Node Status; Table S9: 1-year and 5-year Survival Percentages by Treatment Modality; Figure S1: Survival Analysis of GINEC cases based on Housing location (A) and Median Annual Household Income (B).
