# Supplementary material for: Predictive Nomogram and Propensity Score Matching in Neuroendocrine Carcinoma of the Tubular Gastrointestinal Tract: A US Population-Based Clinical Outcome Study
Source: Cancers (Basel). 2024 May 24;16(11):1998. doi: 10.3390/cancers16111998 (PMC11171326; doi:10.3390/cancers16111998)

Table S1: Overall Survival and Cause Specific Survival Percentages

	1-year Survival (95% C.I)	5-year Survival (95% C.I)
**Overall Survival**	**72.9% (72.0% - 73.7%)**	**54.7% (53.7% - 55.7%)**
**Cause-Specific Survival**	74.2% (73.3% - 75.2%)	60.0% (59.0% - 61.1%)

Table S2: 1-year and 5-year Survival Percentages by Gender

Sex	1-year Survival (95% C.I)	5-year Survival (95% C.I)
**Females**	76.9% (75.7% - 78.1%)	59.0% (57.6% - 60.5%)
**Males**	69.1% (67.9% - 70.4%)	50.7% (49.3% - 52.1%)

Table S3: 1-year and 5-year Survival Percentages by Race

Race	1-year Survival (95% C.I)	5-year Survival (95% C.I)
**White**	70.9% (69.8% - 72.0%)	52.6% (51.3% - 53.8%)
**Black**	77.6% (75.5% - 79.8%)	59.7% (57.1% - 62.4%)
**Hispanic**	74.7% (72.3% - 77.2%)	56.5% (52.6% - 59.5%)
**Asian**	74.8% (71.3% - 78.4%)	56.0% (51.9% - 60.4%)

Table S4: 1-year and 5-year Survival Percentages by Age

Age	1-year Survival (95% C.I)	5-year Survival (95% C.I)
**< 65 years old**	81.7% (80.7% - 82.8%)	68.2% (66.9% - 69.5%)
**≥ 65 years old**	62.4% (61.0% - 63.8%)	38.9% (37.4% - 40.4%)

Table S5: 1-year and 5-year Survival Percentages by Tumor Stage

Tumor Stage	1-year Survival (95% C.I)	5-year Survival (95% C.I)
**Localized**	92.4% (91.5% - 93.4%)	79.9% (78.4% - 81.4%)
**Regional**	82.3% (80.8% - 83.8%)	63.2% (61.3% - 65.2%)
**Distant**	46.1% (44.5% - 47.8%)	24.0% (22.5% - 25.5%)

Table S6: 1-year and 5-year Survival Percentages by Tumor Size

Tumor Size	1-year Survival (95% C.I)	5-year Survival (95% C.I)
**< 2 cm**	93.1% (92.1% - 94.2%)	79.8% (78.2% - 81.5%)
**2 cm – 4 cm**	81.4% (79.5% - 83.4%)	60.8% (58.5% - 63.3%)
**> 4 cm**	50.3% (47.6% - 53.2%)	27.6% (25.2% - 30.2%)

Table S7: 1-year and 5-year Survival Percentages by Tumor Site

Tumor Site	1-year Survival (95% C.I)	5-year Survival (95% C.I)
**Esophagus**	31.1% (26.3% - 36.7%)	7.5% (4.9% - 11.6%)
**Small intestine**	88.7% (87.6% - 89.7%)	69.8% (68.2 – 71.4%)
**Ascending Colon**	42.5% (38.2% - 47.3%)	24.2% (20.5% - 28.6%)
**Transverse Colon**	38.8% (31.6% - 47.7%)	13.6% (8.9% - 20.8%)
**Descending Colon**	28.6% (19.7% - 41.7%)	23.7% (15.4% - 36.5%)
**Sigmoid Colon**	52.3% (46.4% - 59.0%)	39.9% (34.0% - 46.7%)

**Rectum**	79.6% (77.8 – 81.5%)	65.5% (63.3% - 67.8%)
**Appendix**	93.7% (91.8% - 95.5%)	86.5% (83.8% - 89.4%)
**Stomach**	65.5% (63.2% – 67.9%)	43.5% (40.7% - 46.0%)

Table S8: 1-year and 5-year Survival Percentages by Regional Lymph Node Status

**Nodal Status**	**1-year Survival (95% C.I)**	**5-year Survival (95% C.I)**
**Negative**	87.5% (85.5% - 89.5%)	65.7% (62.7% - 68.8%)
**Positive**	76.3% (74.9% - 77.8%)	57.2% (55.4% - 58.9%)

Table S9: 1-year and 5-year Survival Percentages by Treatment Modality

**Treatment**	**1-year Survival (95% C.I)**	**5-year Survival (95% C.I)**
**Chemotherapy only**	34.9% (32.3% - 37.8%)	8.0% (6.4% - 10.0%)
**Surgery only**	87.3% (86.5% - 88.2%)	73.2% (72.0% - 74.4%)
**Chemotherapy and Surgery**	65.3% (62.1% - 68.7%)	33.6% (30.3% - 37.2%)
**None**	56.1% (54.0% - 58.3%)	39.4% (37.1% - 41.7%)

Figure S1: Survival Analysis of GINEC cases based on Housing location (A) and Median Annual Household Income (B)

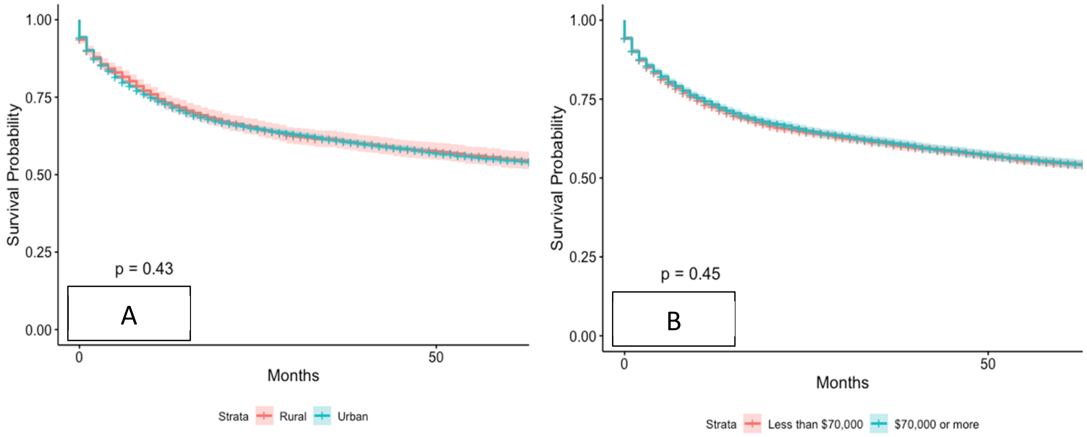

Supplement: Supplementary file 1 [file cancers-16-01998-s001.zip › cancers-2991107-supplementary.pdf]
